# *Saccharomyces cerevisiae* as a model to study synthetic cannabinoids: the impact of using different carbon sources

**DOI:** 10.1080/07853890.2021.1897429

**Published:** 2021-09-28

**Authors:** Daniela Guerreiro, Carla Ferreira, Madalena Salema-Oom, Alexandre Quintas

**Affiliations:** a Instituto Universitário Egas Moniz (IUEM), Egas Moniz Cooperativa de Ensino Superior, Caparica, Portugal; b LabForSci, Centro de Investigação Interdisciplinar Egas Moniz (CiiEM), Egas Moniz Cooperativa de Ensino Superior, Caparica, Portugal; c Laboratório de Ciências Forenses e Psicológicas Egas Moniz, Caparica, Portugal; d UCIBIO, REQUIMTE, Faculdade de Ciências e Tecnologia, Universidade Nova de Lisboa, Caparica, Portugal

## Abstract

**Introduction:**

Synthetic cannabinoids (SC) are potent agonists of cannabinoid receptors, that mime the psychoactive effect of Δ^9^-tetrahydrocannabinol (Δ^9^-THC), the principal psychoactive component of cannabis [1]. JWH-018 was the first novel psychoactive SC found in the recreational drug marketplace in 2008. A few years later, JWH-018 started to be controlled by authorities, so alternative molecules started to emerge. THJ-018 was designed to replace JWH-018, having a similar structural skeleton and also a naphthalene and pentyl chain connected *via* a middle core substructure. Eventually, THJ-018 was scheduled and alternatives emerged, such as EG-018 [2]. This practice makes almost impossible to characterise SC toxicological profiles on an acceptable time scale, mostly due to the time-consuming experiments that must be held in animal models or human cells by standard methods. The yeast *Saccharomyces cerevisiae* shares highly conserved molecular and cellular mechanisms with human cells and has been used before for synthetic cathinones [3]. The present work has studied the best carbon source (glucose or galactose) to measure the impact of synthetic cannabinoids on *S. cerevisiae* growth, aiming to develop a method able to profile synthetic cannabinoids toxicity in a short time scale. The difference between carbon sources is that in Crabtree-positive yeast strains, glucose induce a strong inhibition of mitochondrial oxidative phosphorylation [4].

**Materials and methods:**

The effect of EG-018 was evaluated by its impact on *S. cerevisiae* BY4741 (WT) growth on minimal medium with 2% Glucose or 1% Galactose as sole carbon sources, in the presence and absence of the SC, followed at OD_600nm_. Growth rates at each condition was fitted to a logistic equation.

**Results:**

Figure 1 shows OD_600max_ obtained from the non-linear regression to the logistic equation in the presence of different concentrations of EG-018. While in galactose there is no effect, in 2% Glucose the results points to a growth decrease with EG-018. **Discussion and conclusions:** Comparing the two carbon sources, yeast growth on glucose is more susceptible to EG-018. Recent studies points that JWH-018, a similar SC, exerts an effect on glycolytic and pentose phosphate pathway at high concentrations of glucose (personal communication). As yeast growth in galactose proceeds simultaneously *via* respiration and fermentation due to the “Crabtree effect” [4] an impact of EG-018 on glycolysis might be less effective. These results, suggest that glucose is the best carbon source to develop a yeast based toxicity sensor for SC.

**Figure 1.**
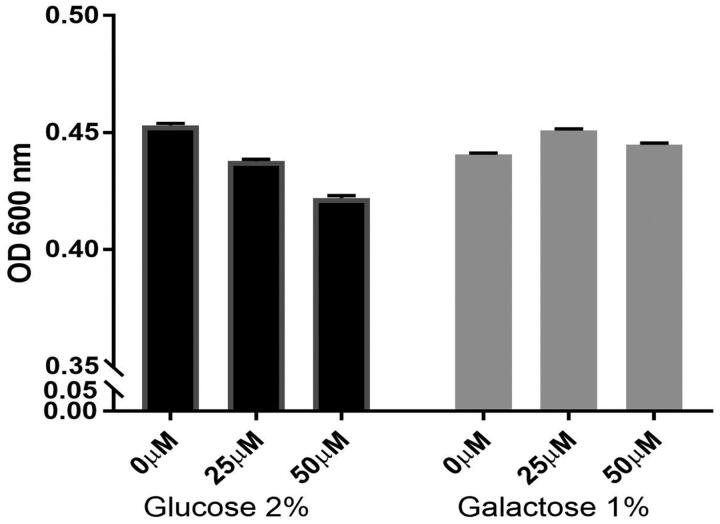
OD_600max_ results in S. cerevisiae BY4741 in the presence of 0, 25 and 50 µM of EG-018 in glucose and galactose.
